# Supplementary material for: The copy-number and varied strengths of MELT motifs in Spc105 balance the strength and responsiveness of the spindle assembly checkpoint
Source: eLife. 2020 Jun 1;9:e55096. doi: 10.7554/eLife.55096 (PMC7292645; doi:10.7554/eLife.55096)
Supplement: Supplementary file 1. [file elife-55096-supp1.docx]

Supplementary file 1: Complete dataset for observations of flow cytometry assay presented in figure 1D.

| Strain | Treatment | Time(hours) | Total number of cells counted | Fraction of cells/population | | |
| --- | --- | --- | --- | --- | --- | --- |
|  |  |  |  | 1N | 2N | 4N |
| WT | DMSO | 0 | 10661 | 0.338711 | 0.541225 | 0.120064 |
|  |  | 1 | 10678 | 0.333677 | 0.553662 | 0.112662 |
|  |  | 2 | 10755 | 0.344305 | 0.538354 | 0.117341 |
|  |  | 3 | 10950 | 0.387306 | 0.495799 | 0.116895 |
|  |  | 4 | 11111 | 0.376294 | 0.493925 | 0.129781 |
| WT | Noc | 0 | 10661 | 0.338711 | 0.541225 | 0.120064 |
|  |  | 1 | 10562 | 0.064382 | 0.762545 | 0.173073 |
|  |  | 2 | 10621 | 0.010074 | 0.771961 | 0.217964 |
|  |  | 3 | 10401 | 0.008076 | 0.735314 | 0.25661 |
|  |  | 4 | 10359 | 0.022396 | 0.631914 | 0.34569 |
| #2 | DMSO | 0 | 10210 | 0.390695 | 0.431244 | 0.178061 |
|  |  | 1 | 9491 | 0.311348 | 0.516173 | 0.172479 |
|  |  | 2 | 9991 | 0.259233 | 0.545991 | 0.194775 |
|  |  | 3 | 9952 | 0.213023 | 0.594252 | 0.192725 |
|  |  | 4 | 10035 | 0.270453 | 0.570703 | 0.158844 |
| #2 | Noc | 0 | 10210 | 0.390695 | 0.431244 | 0.178061 |
|  |  | 1 | 10030 | 0.264506 | 0.536391 | 0.199103 |
|  |  | 2 | 10013 | 0.136023 | 0.542595 | 0.321382 |
|  |  | 3 | 9883 | 0.091774 | 0.569766 | 0.33846 |
|  |  | 4 | 9973 | 0.068284 | 0.556202 | 0.375514 |
| #4 | DMSO | 0 | 9810 | 0.281142 | 0.524669 | 0.19419 |
|  |  | 1 | 9916 | 0.274506 | 0.544776 | 0.180718 |
|  |  | 2 | 9921 | 0.282129 | 0.557706 | 0.160165 |
|  |  | 3 | 9991 | 0.258633 | 0.597938 | 0.143429 |
|  |  | 4 | 9915 | 0.232779 | 0.597176 | 0.170045 |
| #4 | Noc | 0 | 9810 | 0.281142 | 0.524669 | 0.19419 |
|  |  | 1 | 9700 | 0.114227 | 0.598763 | 0.28701 |
|  |  | 2 | 9680 | 0.059814 | 0.532438 | 0.407748 |
|  |  | 3 | 9664 | 0.042425 | 0.451159 | 0.506416 |
|  |  | 4 | 9569 | 0.072003 | 0.334204 | 0.593792 |
| #6 | DMSO | 0 | 10244 | 0.223057 | 0.596251 | 0.180691 |
|  |  | 1 | 10458 | 0.148021 | 0.634729 | 0.21725 |
|  |  | 2 | 10207 | 0.151661 | 0.585481 | 0.262859 |
|  |  | 3 | 10260 | 0.162671 | 0.640253 | 0.197076 |
|  |  | 4 | 10114 | 0.162745 | 0.666205 | 0.17105 |
| #6 | Noc | 0 | 10244 | 0.223057 | 0.596251 | 0.180691 |
|  |  | 1 | 9978 | 0.055823 | 0.664662 | 0.279515 |
|  |  | 2 | 9897 | 0.041831 | 0.630696 | 0.327473 |
|  |  | 3 | 9929 | 0.04361 | 0.620707 | 0.335683 |
|  |  | 4 | 10089 | 0.061552 | 0.561205 | 0.377243 |
| #4^MELT^ | DMSO | 0 | 10467 | 0.386071 | 0.518869 | 0.095061 |
|  |  | 1 | 10396 | 0.302713 | 0.591477 | 0.10581 |
|  |  | 2 | 10588 | 0.34813 | 0.550057 | 0.101813 |
|  |  | 3 | 10788 | 0.317946 | 0.558027 | 0.124027 |
|  |  | 4 | 10808 | 0.417283 | 0.484178 | 0.098538 |
| #4^MELT^ | Noc | 0 | 10467 | 0.386071 | 0.518869 | 0.095061 |
|  |  | 1 | 10317 | 0.065232 | 0.807599 | 0.127169 |
|  |  | 2 | 10162 | 0.015843 | 0.767664 | 0.216493 |
|  |  | 3 | 9964 | 0.018065 | 0.749297 | 0.232637 |
|  |  | 4 | 9498 | 0.035902 | 0.725311 | 0.238787 |
| Spc105-6A | DMSO | 0 | 10015 | 0.445032 | 0.474089 | 0.080879 |
|  |  | 1 | 10375 | 0.253494 | 0.532916 | 0.21359 |
|  |  | 2 | 10356 | 0.236868 | 0.542487 | 0.220645 |
|  |  | 3 | 10307 | 0.221597 | 0.532842 | 0.245561 |
|  |  | 4 | 10314 | 0.22804 | 0.547605 | 0.224355 |
| Spc105-6A | Noc | 0 | 10245 | 0.437677 | 0.451733 | 0.110591 |
|  |  | 1 | 10487 | 0.194717 | 0.613045 | 0.192238 |
|  |  | 2 | 10286 | 0.112969 | 0.517402 | 0.369629 |
|  |  | 3 | 10309 | 0.096906 | 0.369871 | 0.533223 |
|  |  | 4 | 10276 | 0.097509 | 0.276761 | 0.62573 |
