# Supplementary material for: The copy-number and varied strengths of MELT motifs in Spc105 balance the strength and responsiveness of the spindle assembly checkpoint
Source: eLife. 2020 Jun 1;9:e55096. doi: 10.7554/eLife.55096 (PMC7292645; doi:10.7554/eLife.55096)
Supplement: Supplementary file 2. [file elife-55096-supp2.docx]

Supplementary file 2: Strains used in this study

| Strain (AJY#) | Background | Genotype | Used in figure |
| --- | --- | --- | --- |
| 4650 | YEF473 | *MATa, spc105Δ::NAT, leu2Δ-1::Spc105^222::GFP^ (LEU2), BUB1-ymCherry-HYG* | 1C, 2A, 5A-B, 5-FIG SUPP1A-B |
| 5709 | YEF473 | *MATα, spc105Δ::NAT*, *leu2Δ-1::*Spc105-5A^101-104::AAAA^ *(T149A, T211A, T235A, T284A, T313A)*-*12XMYC* (*LEU2*), *SPC25-GFP-HIS3*, *BUB1-ymCherry-HYG* | 1C |
| 5710 | YEF473 | *MATa, spc105Δ::NAT*, *leu2Δ-1::*Spc105-5A^101-104::AAAA^ *(T149A, T172A, T211A, T284A, T313A)*-*12MYC* (*LEU2*), *SPC25-GFP-HIS3*, *BUB1-ymCherry-HYG* | 1C |
| 5711 | YEF473 | *MATa, spc105Δ::NAT*, *leu2Δ-1::*Spc105-5A^101-104::AAAA^ *(T149A, T172A, T211A, T284A, T313A)*-1*2XMYC* (*LEU2*), *SPC25-GFP-HIS3*, *BUB1-ymCherry-HYG* | 1C |
| 5712 | YEF473 | *MATa, spc105Δ::NAT*, *leu2Δ-1::*Spc105-5A^101-104::AAAA^ *(T149A, T172A, T211A, T235A, T284A)*-*12XMYC* (*LEU2*), *SPC25-GFP-HIS3*, *BUB1-ymCherry-HYG* | 1C |
| 5608 | YEF473 | *MATa, spc105Δ::NAT, leu2Δ-1::Spc105-5A (T149A, T172A, T211A, ID232-233ME, T284A, T313A)^222::GFP, 101-104, 340-343::AAAA^ (LEU2), BUB1-mCherry-HYG* | 1C |
| 5609 | YEF473 | *MATa, spc105Δ::NAT, leu2Δ-1::Spc105-5A (T149A, T172A, T211A, ID232-233ME, T284A, T313A)^222::GFP, 101-104, 340-343::AAAA^ (LEU2), BUB1-mCherry-HYG* | 1C |
| 3133 | YEF473 | *MATa, spc105Δ::NAT, leu2Δ-1::* Spc105^222::GFP^ *(LEU2)* | 1D,2C-E, 4C, 6C-D, 1-FIG SUPP1, 2-FIG SUPP1C-F, 2-FIG SUPP 2B, 3-FIG SUPP 1D, 5-FIG SUPP1F |
| 4022 | YEF473 | *MATa, spc105Δ::NAT, leu2Δ-1::* Spc105-6A *(T149A, T172A, T211A, T235A, T284A, T313A)*-*12XMYC (LEU2)* | 1D, 2D-E, 1-FIG SUPP1, 2-FIG SUPP 1C, 2-FIG SUPP 3B, |
| 4054 | YEF473 | *MATa, spc105Δ::NAT, leu2Δ-1::Spc105-5A^101-104::AAAA^ (T149A, T211A, T235A, T284A, T313A)-12XMYC (LEU2)* | 1D, 1-FIG SUPP1, 2-FIG SUPP 1C-D, |
| 4055 | YEF473 | *MATα, spc105Δ::NAT, leu2Δ-1::Spc105-5A^101-104::AAAA^ (T149A, T172A, T211A, T284A, T313A)-12XMYC (LEU2)* | 1D, 1-FIG SUPP1, 2-FIG SUPP 1C-D |
| 4056 | YEF473 | *MATα, spc105Δ::NAT, leu2Δ-1::Spc105-5A^101-104::^*^AAAA^ *(T149A, T172A, T211A, T235A, T284A)-12XMYC (LEU2)* | 1D, 1-FIG SUPP1, 2-FIG SUPP 1C |
| 4058 | YEF473 | *MATa, spc105Δ::NAT, leu2Δ-1::Spc105-5A^101-104::AAAA^ (T149A, T211A, T235A, T284A, T313A)-12XMYC (LEU2)* | 1D, 1-FIG SUPP1, 2-FIG SUPP 1C-D |
| 4059 | YEF473 | *MATa, spc105Δ::NAT, leu2Δ-1::Spc105-5A^101-104::AAAA^ (T149A, T172A, T211A, T284A, T313A)-12XMYC (LEU2)* | 1D, 1-FIG SUPP1, 2-FIG SUPP 1C-D |
| 4060 | YEF473 | *MATa, spc105Δ::NAT, leu2Δ-1::Spc105-5A^101-104::AAAA^ (T149A, T172A, T211A, T235A, T284A)-12XMYC (LEU2)* | 1D, 1-FIG SUPP1, 2-FIG SUPP 1C-D |
| 5606 | YEF473 | *MATa, spc105Δ::NAT, leu2Δ-1::Spc105-5A (T149A, T172A, T211A, ID232-233ME, T284A, T313A)^222::GFP, 101-104, 340-343::AAAA^ (LEU2)* | 1D, 1-FIG SUPP1, 2-FIG SUPP1D |
| 5607 | YEF473 | *MATα, spc105Δ::NAT, leu2Δ-1::Spc105-5A (T149A, T172A, T211A, ID232-233ME, T284A, T313A)^222::GFP, 101-104, 340-343::AAAA^ (LEU2)* | 1D, 2-FIG SUPP 1D |
| 5199 | YEF473 | *MATa, TetR-GFP (LEU2); SPC29-mCherry-HIS3, TetO-CENIV (URA3), spc105Δ::TRP1::*Spc105^222::mCherry^ *(KAN)* | 2A |
| 5736 | YEF473 | *MATa, spc105Δ::NAT, leu2Δ-1::Spc105^222::GFP^ (LEU2), SPC97-mCherry-HYG, ura3-52::prGAL1-CIK1-CC (URA3)* | 2B, 2-FIG SUPP 1B, 2-FIG SUPP 2B |
| 5762 | YEF473 | *MATa, spc105Δ::NAT, leu2Δ-1::Spc105-4A* (T149A, T211A, T235A, T284A)*-12XMYC (LEU2), NSL1-GFP-HIS3, ura3-52::prGAL1-CIK1-CC (URA3), SPC72-mCherry-HYG* | 2B, 2-FIG SUPP 1B, 2-FIG SUPP 2B |
| 5763 | YEF473 | *MATa, spc105Δ::NAT, leu2Δ-1::Spc105-4A* (T149A, T211A, T235A, T284A)*-12XMYC (LEU2), NSL1-GFP-HIS3, ura3-52::prGAL1-CIK1-CC (URA3), SPC72-mCherry-HYG* | 2B, 2-FIG SUPP 1B, 2-FIG SUPP 2B |
| 4016 | YEF473 | *MATa, spc105Δ::NAT, leu2Δ-1::Spc105-5A (T149A, T211A, T235A, T284A, T313A)-12MYC (LEU2)* | 2C-D |
| 4057 | YEF473 | *MATα, spc105Δ::NAT, leu2Δ-1::Spc105-4A (T149A, T211A, T235A, T284A)-12XMYC (LEU2)* | 2C-D |
| 4133 | YEF473 | *MATα, spc105Δ::NAT, leu2Δ-1::Spc105-4A^222::GFP^ (T211A, T235A, T284A, T313A)(LEU2)* | 2C-D |
| 5603 | YEF473 | *MATa, spc105Δ::NAT, leu2Δ-1::Spc105-5A^222::GFP^ (T149A, M171L, T211A, T235A, T284A, T313A)(LEU2)* | 2-FIG SUPP1D |
| 5604 | YEF473 | *MATα, spc105Δ::NAT, leu2Δ-1::Spc105-5A^222::GFP^ (T149A, M171L, T211A, T235A, T284A, T313A)(LEU2)* | 2-FIG SUPP1D |
| 4134 | YEF473 | *MATα, spc105Δ::NAT, leu2Δ-1::Spc105-4A^222::GFP^ (T211A, T235A, T284A, T313A)(LEU2)* | 2D |
| 4135 | YEF473 | *MATa, spc105Δ::NAT, leu2Δ-1::Spc105-4A^222::GFP^ (T172A, T235A, T284A, T313A)(LEU2)* | 2D |
| 4136 | YEF473 | *MATa, spc105Δ::NAT, leu2Δ-1::Spc105-4A^222::GFP^ (T172A, T235A, T284A, T313A)(LEU2)* | 2D |
| 4065 | YEF473 | *MATa, spc105Δ::NAT, leu2Δ-1::Spc105-3A (T149A, T172A, T211A) ^222::GFP^ (LEU2)* | 2C-E, 6C, 2-FIG SUPP 3B, 5-FIG SUPP1F |
| 4101 | YEF473 | *spc105Δ::NAT, leu2Δ-1::Spc105-3A (T149A, T172A, T211A)^222::GFP^ (LEU2)* | 2D |
| 4066 | YEF473 | *MATα, spc105Δ::NAT, leu2Δ-1::Spc105-3A (*T235A, T284A, T313A*)^222::GFP^ (LEU2)* | 2C-E |
| 4102 | YEF473 | *spc105Δ::NAT, leu2Δ-1::Spc105-3A (T235A, T284A, T313A) ^222::GFP^ (LEU2)* | 2D |
| 4680 | YEF473 | *MATa, spc105Δ::NAT, leu2Δ-1::Spc105-3A (T149A, T172A, T211A, #1-3 in #4-6)^222::GFP^ (LEU2)* | 2E |
| 4681 | YEF473 | *MATα, spc105Δ::NAT, leu2Δ-1::Spc105-3A (T149A, T172A, T211A, #1-3 in #4-6)^222::GFP^ (LEU2)* | 2E |
| 4642 | YEF473 | *MATa, spc105Δ::NAT, leu2Δ-1::Spc105-3A (#4-6 in #1-3, T235A, T284A, T313A)^222::GFP^ (LEU2)* | 2E |
| 4643 | YEF473 | *MATα, spc105Δ::NAT, leu2Δ-1::Spc105-3A (#4-6 in #1-3, T235A, T284A, T313A) ^222::GFP^ (LEU2)* | 2E |
| 4252 | YEF473 | *spc105Δ::NAT, leu2Δ-1::Spc105-5A (T149A, T172A, T211A, T235A, T284A, ME310-311ID)^101-104::AAAA^ (LEU2)* | 2-FIG SUPP 1C |
| 4253 | YEF473 | *spc105Δ::NAT, leu2Δ-1::Spc105-5A (T149A, T172A, T211A, T235A, T284A, ME310-311ID)^101-104::AAAA^ (LEU2)* | 2-FIG SUPP 1C |
| 5951 | W303 | *MATa, ade2-1 ura3 trp1-1 lys2 his3 CFIII(CEN3.L.YPH278)URA3-SUP11, spc105Δ::NAT, Spc105 ^222::mCherry^ (LEU2)* | 2F, 6D |
| 3569 | W303 | *MATa, ade2-1 ura3-1 leu2Δ-1 trp1-1 lys2-801 his3 CFIII(CEN3.L.YPH278)URA3-SUP11, mad1Δ::HIS3* | 2F, 6D |
| 5991 | W303 | *MATa, ade2-1 ura3 trp1-1 lys2 his3 CFIII(CEN3.L.YPH278)URA3-SUP11, spc105Δ::NAT, leu2Δ-1::Spc105-3A (T149A, T172A, T211A)^222::GFP^ (LEU2)* | 2F, 6D |
| 3357 | YEF473 | *MATα, fpr1Δ, NDC80-FKBP12-HIS3, leu2Δ-1::prHis3-GFP-Spc105^120-329^-FRB (LEU2), BUB3-mCherry-HYG* | 3A-B, 3-FIG SUPP 1C |
| 3889 | YEF473 | *MATα, fpr1Δ, NDC80-FKBP12-HIS3, leu2Δ-1::prHIS3-GFP(S65T)- Spc105^2-329^-FRB(LEU2), BUB3-mCherry-HYG* | 3A-B, 3-FIG SUPP 1C |
| 3890 | YEF473 | *MATα, fpr1Δ, NDC80-FKBP12-HIS3, leu2Δ-1::prHIS3-GFP(S65T)- Spc105^2-329^-FRB (LEU2), BUB3-mCherry-Hyg* | 3A-B, 3-FIG SUPP 1C |
| 3936 | YEF473 | *MATa, fpr1Δ, MTW1-FKBP12-HIS3, leu2Δ-1::prHIS3-GFP(S65T)- Spc105^120-329^-FRB (LEU2), BUB3-mCherry-Hyg* | 3A-B, 3-FIG SUPP 1C |
| 3937 | YEF473 | *MATa, fpr1Δ, MTW1-FKBP12-His3, leu2Δ-1::prHIS3-GFP(S65T)- Spc105^120-329^-FRB (LEU2), BUB3-mCherry-Hyg* | 3A-B, 3-FIG SUPP 1C |
| 3891 | YEF473 | *MATα, fpr1Δ, MTW1-FKBP12-HIS3, leu2Δ-1::prHIS3-GFP(S65T)- Spc105^2-329^-FRB (LEU2), BUB3-mCherry-Hyg* | 3A-B, 3-FIG SUPP 1C |
| 3892 | YEF473 | *MATα, fpr1Δ, MTW1-FKBP12-HIS3, leu2Δ-1::prHIS3-GFP(S65T)-Spc105^2-329^-FRB (LEU2), BUB3-mCherry-HYG* | 3A-B, 3-FIG SUPP 1C |
| 3941 | YEF473 | *MATa, fpr1Δ, SPC24-2XFKBP12-HIS3, BUB3-mCherry-HYG, leu2Δ-1::prHIS3-GFP(S65T)-Spc105^120-329^-FRB (LEU2)* | 3A-B, 3-FIG SUPP 1C |
| 3942 | YEF473 | *MATa, fpr1Δ, SPC24-2XFKBP12-HIS3, BUB3-mCherry-HYG, leu2Δ-1::prHIS3-GFP(S65T)- Spc105^2-329^-FRB (LEU2)* | 3A-B, 3-FIG SUPP 1C |
| 3371 | YEF473 | *MATa, fpr1Δ, BUB3-mCherry-HYG, leu2Δ-1::prHIS3-GFP(S65T)- Spc105^120-329^-FRB (LEU2), SPC34-2xFKBP12:HIS3* | 3A-B |
| 3606 | YEF473 | *MATa, spc105Δ::NAT, leu2Δ-1::Spc105^222::GFP^ (LEU2), BUB3-mCherry-HYG* | 3A, 4B, 6B, 5-FIG SUPP1D |
| 3360 | YEF473 | *MATα, fpr1Δ, NDC80-FKBP12-HIS3, leu2Δ-1::prHIS3-GFP- Spc105^120-329^-FRB (LEU2), BUB3-mCherry-HYG, prMET3-CDC20 (TRP1)* | 3C |
| 5035 | YEF473 | *MATα, fpr1Δ, NDC80-FKBP-HIS3, leu2Δ-1::prHIS3-GFP(S65T)- Spc105^120-329^-FRB (LEU2), MAD1-mCherry-Hyg, nup60Δ::TRP1* | 3D, 6F |
| 2897 | BY4742 | *MATα, fpr1Δ, ASK1-2xFKBP12-HIS3, leu2Δ-1::prHIS3-GFP-Spc105^120-329^-FRB (LEU2), MAD1-mCherry-Hyg, nup60Δ::TRP1* | 3D, 6F, 3-FIG SUPP 1B |
| 4968 | YEF473 | *MATa, spc105Δ::NAT1, leu2Δ-1::Spc105 RASA (V76, F78::A)^222::GFP^ (LEU2), mad2Δ::TRP1, BUB3-mCherry-HYG* | 4B, 5-FIG SUPP1D |
| 4969 | YEF473 | *MATa, spc105Δ::NAT1, leu2Δ-1::Spc105 RASA (V76, F78::A)^222::GFP^ (LEU2), mad2Δ::TRP1, BUB3-mCherry-HYG* | 4B |
| 3781 | YEF473 | *MATa, spc105Δ::NAT, leu2Δ-1::Spc105^222::GFP^ (LEU2), nup60Δ::TRP1, MAD1-mcherry-HYG* | 4B-C, 5-FIG SUPP1E |
| 5633 | YEF473 | *MATα, mad3Δ::lox-URA3-lox, MAD1-mCherry-HYG, nup60Δ::TRP1, spc105Δ::NAT, leu2Δ-1::Spc105 RASA (V76, F78::A)^222::GFP^ (LEU2)* | 4B |
| 5634 | YEF473 | *MATα, mad3Δ::lox-URA3-lox, MAD1-mCherry-HYG, nup60Δ::TRP1, spc105Δ::NAT, leu2Δ-1::Spc105 RASA (V76, F78::A)^222::GFP^ (LEU2)* | 4B |
| 4926 | YEF473 | *MAT a/α, spc105Δ::NAT/SPC105, ura3-52::Spc105 RASA (V76, F78::A)^222::GFP^ (URA3)* | 4C |
| 5803 | YEF473 | *MATa/α, SPC105/spc105Δ::NAT, ura3-52/ura3-52::Spc105 RASA (V76, F78::A)^222::GFP^ (URA3), BUB1/bub1Δ::TRP1::bub1-T453A-T455A-2XFKBP12 (KAN)* | 4C |
| 5713 | YEF473 | *MATa, bub1Δ::TRP1::bub1-T455A,T485A-2XFKBP12 (KAN), MAD1-mCherry-HYG, nup60Δ::TRP1, SPC25-GFP-HIS3* | 4C |
| 5337 | YEF473 | *fpr1Δ, bub1Δ::TRP1* | 4C |
| 5893 | YEF473 | *bub1-T453A, T455A-2XFKBP12 (KAN)* | 4C |
| 5894 | YEF473 | *bub1-T453A, T455A-2XFKBP12 (KAN)* | 4C |
| 5895 | YEF473 | *spc105Δ::NAT, ura3-52::Spc105 RASA (V76, F78::A)^222::GFP^ (URA3), bub1-T453A, T455A-2XFKBP12 (KAN)* | 4C, 3-FIG SUPP 1D |
| 5896 | YEF473 | *spc105Δ::NAT, ura3-52::Spc105 RASA (V76, F78::A)^222::GFP^ (URA3), bub1-T453A, T455A-2XFKBP12 (KAN)* | 4C |
| 5866 | YEF473 | *MATa, spc105Δ::NAT, leu2Δ-1::* *Spc105 MELT1(X6)^455::GFP^ (LEU2), BUB1-ymCherry-HYG* | 5A-B 5-FIG SUPP1A-B |
| 5867 | YEF473 | *MATa, spc105Δ::NAT, leu2Δ-1::* *Spc105 MELT1(X6)^455::GFP^ (LEU2), BUB1-ymCherry-HYG* | 5A |
| 5887 | YEF473 | *MATa, spc105Δ::NAT, leu2Δ-1::* *Spc105 MELT1(X6)^455::GFP^ (LEU2), BUB1-ymCherry-HYG, mad1Δ::TRP1* | 5A |
| 5888 | YEF473 | *MATa, spc105Δ::NAT, leu2Δ-1::* *Spc105 MELT1(X6)^455::GFP^ (LEU2), BUB1-ymCherry-HYG, mad1Δ::TRP1* | 5A |
| 5904 | YEF473 | *MATa, spc105Δ::NAT, leu2Δ-1::nSpc105^222::GFP^ (LEU2), (KAN)-prGAL1-BUB1-ymCherry-HYG* | 5B |
| 5905 | YEF473 | *MATa, spc105Δ::NAT, leu2Δ-1::* *Spc105 MELT1(X6)^455::GFP^ (LEU2), (KAN)-prGAL1-BUB1-ymCherry-HYG* | 5B, 5-FIG SUPP1B |
| 5906 | YEF473 | *MATa, spc105Δ::NAT, leu2Δ-1::* *Spc105 MELT1(X6)^455::GFP^ (LEU2), (KAN)-prGAL1-BUB1-ymCherry-HYG* | 5B, 5-FIG SUPP1B |
| 5013 | YEF473 | *MATa/α, spc105Δ::NAT/SPC105, leu2Δ-1/leu2Δ-1::Spc105-3A RASA (V76, F78::A)^222::GFP^ (T149A, T172A, T211A) (LEU2)* | 6A |
| 5014 | YEF473 | *MATa/α,spc105Δ::NAT/SPC105, leu2Δ-1/leu2Δ-1::Spc105-3A RASA (V76, F78::A)^222::GFP^ (T235A, T284A, T313A) (LEU2)* | 6A |
| 5074 | YEF473 | *MATa, spc105Δ::NAT, leu2Δ-1::Spc105-3A (T149A, T172A, T211A) RASA (V76, F78::A)^222::GFP^ (LEU2), BUB3-mCherry-HYG* | 6B |
| 5075 | YEF473 | *MATa, spc105Δ::NAT, leu2Δ-1::Spc105-3A (T149A, T172A, T211A) RASA (V76, F78::A)^222::GFP^ (LEU2), BUB3-mCherry-HYG* | 6B |
| 5052 | YEF473 | *MATa, spc105Δ::NAT, leu2Δ-1::Spc105-3A RASA (V76, F78::A)^222::GFP^ (T149A, T172A, T211A) (LEU2)* | 5C-D |
| 5053 | YEF473 | *MATα, spc105Δ::NAT, leu2Δ-1::Spc105-3A RASA (V76, F78::A)^222::GFP^ (T149A, T172A, T211A) (LEU2)* | 5C-D, 5-FIG SUPP1F |
| 5989 | W303 | *MATa, ade2-1 ura3 trp1-1 lys2 his3 CFIII(CEN3.L.YPH278)URA3-SUP11, spc105Δ::NAT, leu2Δ-1::Spc105-3A (T149A, T172A, T211A) RASA (V76, F78::A)^222::GFP^ (LEU2)* | 6E |
| 5990 | W303 | *MATa, ade2-1 ura3 trp1-1 lys2 his3 CFIII(CEN3.L.YPH278)URA3-SUP11, spc105Δ::NAT, leu2Δ-1::Spc105-3A (T149A, T172A, T211A) RASA (V76, F78::A)^222::GFP^ (LEU2)* | 6E |
| 5987 | YEF473 | *fpr1Δ, NDC80-FKBP12-HIS3, leu2Δ-1::prHIS3-GFP-Spc105^120-329^-FRB (LEU2), Mad1-mCherry-HYG, nup60Δ::Trp1, Spc105^222::GFP^ RASA (V76, F78::A) (T149A, T172A, T211A) (URA3), spc105Δ::NAT* | 6F, 5-FIG SUPP1G |
| 5988 | YEF473 | *fpr1Δ, NDC80-FKBP12-HIS3, leu2Δ-1::prHIS3-GFP- Spc105^120-329^-FRB (LEU2), Mad1-mCherry-Hyg, nup60Δ::Trp1, Spc105^222::GFP^ RASA (V76, F78::A) (T149A, T172A, T211A) (URA3), spc105Δ::NAT* | 6F, 5-FIG SUPP1G |
| 2787 | BY4742 | *MATα, fpr1Δ, ASK1-2xFKBP12-HIS3, leu2Δ-1::prHIS3-GFP-Spc105^120-329^-FRB (LEU2)* | 6F |
| 5610 | YEF473 | *MATa, spc105Δ::NAT, leu2Δ-1::Spc105-5A (T149A, T172A, T211A, T235A, T313A)^222::GFP, 101-104, 340-343::AAAA^ (LEU2)* | 2-FIG SUPP 1D |
| 5611 | YEF473 | *MATa, spc105Δ::NAT, leu2Δ-1::Spc105-5A (T149A, T172A, T211A, T235A, T313A)^222::GFP, 101-104, 340-343::AAAA^ (LEU2)* | 2-FIG SUPP 1D |
| 5614 | YEF473 | *MATa, spc105Δ::NAT, leu2Δ-1::Spc105-5A (T149A, T172A, T211A, T235A, I283L, T313A)^222::GFP, 101-104, 340-343::AAAA^ (LEU2)* | 2-FIG SUPP 1D |
| 5615 | YEF473 | *MATα, spc105Δ::NAT, leu2Δ-1::Spc105-5A (T149A, T172A, T211A, T235A, I283L, T313A)^222::GFP, 101-104, 340-343::AAAA^ (LEU2)* | 2-FIG SUPP 1D |
| 4195 | YEF473 | *MATa, spc105Δ::NAT, leu2Δ-1:: Spc105-3A (T149A, T172A, T211A)^101-104::AAAA, 222::GFP^ (LEU2)* | 2-FIG SUPP 3B |
| 4196 | YEF473 | *MATα, spc105Δ::NAT, leu2Δ-1:: Spc105-3A (T149A, T172A, T211A)^101-104::AAAA, 222::GFP^ (LEU2)* | 2-FIG SUPP 3B |
| 4727 | YEF473 | *MATa, spc105Δ::NAT, leu2Δ-1::Spc105-3A (#4-6 in #1-3, T235A, T284A, T313A)^101-104::AAAA, 222::GFP^ (LEU2)* | 2-FIG SUPP 3B |
| 4728 | YEF473 | *MATa, spc105Δ::NAT, leu2Δ-1::Spc105-3A (#4-6 in #1-3, T235A, T284A, T313A)^101-104::AAA, 222::GFP^ (LEU2)* | 2-FIG SUPP 3B |
| 4729 | YEF473 | *MATa, spc105Δ::NAT, leu2Δ-1::Spc105-3A (T149A, T172A, T211A, #1-3 in #4-6)^101-104::AAAA, 222::GFP^ (LEU2)* | 2-FIG SUPP 3B |
| 4730 | YEF473 | *MATa, spc105Δ::NAT, leu2Δ-1::Spc105-3A (T149A, T172A, T211A,* *#1-3 in #4-6*)*^101-104::AAAA, 222::GFP^ (LEU2)* | 2-FIG SUPP 3B |
| 4476 | YEF473 | *MATa, spc105Δ::NAT, leu2Δ-1::Spc105-3A (T149A, T172A, T211A) ^101-104,340-343::AAAA, 222::GFP^ (LEU2), bub1ΔK::mCherry-HYG* | 2-FIG SUPP 1E |
| 4477 | YEF473 | *MATa, spc105Δ::NAT, leu2Δ-1::Spc105-3A (T149A, T172A, T211A)^101-104,340-343::AAAA 222::GFP^ (LEU2), bub1ΔK::mCherry-HYG* | 2-FIG SUPP 1E |
| 4478 | YEF473 | *MATa, spc105Δ::NAT, leu2Δ-1::Spc105-3A (T235A, T284A, T313A)^101-104,340-343::AAAA, 222::GFP^ (LEU2), bub1ΔK::mCherry-HYG* | 2-FIG SUPP 1E |
| 4479 | YEF473 | *MATa, spc105Δ::NAT, leu2Δ-1::Spc105-3A (T235A, T284A, T313A)^101-104,340-343::AAAA, 222::GFP^ (LEU2), bub1ΔK::mCherry-HYG* | 2-FIG SUPP 1E |
| 4384 | YEF473 | *MATa, spc105Δ::NAT, leu2Δ-1:: Spc105^222::GFP, 101-104, 340-340::AAAA^ (LEU2),* bub1ΔK*-mCherry-HYG* | 2-FIG SUPP 1E |
| 4415 | YEF473 | *MATa, spc105Δ::NAT, leu2Δ-1:: Spc105 ^222::GFP^ (LEU2), bub1ΔK-mCherry-HYG* | 2-FIG SUPP 1E |
| 3635 | YEF473 | *MATa, spc105Δ::NAT, leu2Δ-1::Spc105^222::GFP^ (LEU2), BUB3-mCherry-HYG, prMET3-CDC20 (URA3)* | 2-FIG SUPP 3A |
| 3638 | YEF473 | *MATa, spc105Δ::NAT, leu2Δ-1::Spc105^222::GFP, 101-104,340-343::AAAA^ (LEU2), BUB3-mCherry-HYG, prMET3-CDC20 (URA3)* | 2-FIG SUPP 3A |
| 3909 | YEF473 | *MATa, spc105Δ::NAT, leu2Δ-1::Spc105^222::GFP, 101-104,340-343::AAAA^ T149A, T172A, T211A (LEU2), BUB3-mCherry-HYG, prMET3-CDC20 (URA3)* | 2-FIG SUPP 3A |
| 3910 | YEF473 | *MATa, spc105Δ::NAT, leu2Δ-1::Spc105^222::GFP, 101-104,340-343::AAAA^ T149A, T172A, T211A (LEU2), BUB3-mCherry-HYG, prMET3-CDC20 (URA3)* | 2-FIG SUPP 3A |
| 3911 | YEF473 | *MATa, spc105Δ::NAT, leu2Δ-1::Spc105^222::GFP, 101-104,340-343::AAAA^ T235A, T284A, T313A (LEU2), BUB3-mCherry-HYG, prMET3-CDC20 (URA3)* | 2-FIG SUPP 3A |
| 3912 | YEF473 | *MATα, spc105Δ::NAT, leu2Δ-1::Spc105^222::GFP, 101-104,340-343::AAAA^ T235A, T284A, T313A (LEU2), BUB3-mCherry-HYG, prMET3-CDC20 (URA3)* | 2-FIG SUPP 3A |
| 4749 | YEF473 | *MATa, spc105Δ::NAT, leu2Δ-1::Spc105-3A (#4-6 in #1-3, T235A, T284A, T313A)^101-104::AAAA, 222::GFP^ (LEU2), BUB3-mCherry-HYG* | 2-FIG SUPP 3A |
| 4750 | YEF473 | *MATα, spc105Δ::NAT, leu2Δ-1::Spc105-3A (#4-6 in #1-3, T235A, T284A, T313A)^101-104::AAAA, 222::GFP^ (LEU2), BUB3-mCherry-HYG* | 2-FIG SUPP 3A |
| 4751 | YEF473 | *MATa, spc105Δ::NAT, leu2Δ-1::Spc105-3A (T149A, T172A, T211A, #1-3 in #4-6)^101-104::AAAA, 222::GFP^ (LEU2), BUB3-mCherry-HYG* | 2-FIG SUPP 3A |
| 4752 | YEF473 | *MATα, spc105Δ::NAT, leu2Δ-1::Spc105-3A (T149A, T172A, T211A, #1-3 in #4-6)^101-104::AAAA 222::GFP^ (LEU2), BUB3-mCherry-HYG* | 2-FIG SUPP 3A |
| 4529 | YEF473 | *MATa, spc105Δ::NAT, leu2Δ-1::Spc105-3A (T149A, T172A, T211A) ^101-104::AAAA 222::mCherry^ (LEU2), Sgo1-GFP-HIS3* | 2-FIG SUPP 3D |
| 4547 | YEF473 | *MATa, spc105Δ::NAT, leu2Δ-1::Spc105-3A (T235A, T284A, T313A) ^101-104::AAAA 222::mCherry^ (LEU2), Sgo1-GFP-HIS3* | 2-FIG SUPP 3D |
| 4304 | YEF473 | *MATa, spc105Δ::NAT, leu2Δ-1::Spc105-3A (T149A, T172A, T211A)^222::GFP^ (LEU2), ura3-52::prGAL1-CIK1-CC (URA3)* | 2-FIG SUPP 2A |
| 4305 | YEF473 | *MATa, spc105Δ::NAT, leu2Δ-1::Spc105-3A (T149A, T172A, T211A)^222::GFP^ (LEU2), ura3-52::prGAL1-CIK1-CC (URA3)* | 2-FIG SUPP 2A |
| 4306 | YEF473 | *MATa, spc105Δ::NAT, leu2Δ-1::Spc105-3A (T235A, T284A, T313A)^222::GFP^ (LEU2), ura3-52::prGAL1-CIK1-CC (URA3)* | 2-FIG SUPP 2A |
| 4307 | YEF473 | *MATa, spc105Δ::NAT, leu2Δ-1::Spc105-3A (T235A, T284A, T313A)^222::GFP^ (LEU2), ura3-52::prGAL1-CIK1-CC (URA3)* | 2-FIG SUPP 2A |
| 4308 | YEF473 | *MATa, spc105Δ::NAT, leu2Δ-1::Spc105-3A (T149A, T172A, T211A)^101-104, 340-343::AAAA, 222::GFP^ (LEU2), ura3-52::prGAL1-CIK1-CC (URA3)* | 2-FIG SUPP 2A |
| 4309 | YEF473 | *MATa, spc105Δ::NAT, leu2Δ-1::Spc105-3A (T149A, T172A, T211A)^101-104, 340-343::AAA, 222::GFP^ (LEU2), ura3-52::prGAL1-CIK1-CC (URA3)* | 2-FIG SUPP 2A |
| 4310 | YEF473 | *MATa, spc105Δ::NAT, leu2Δ-1::Spc105-3A (T235A, T284A, T313A)^101-104, 340-343::AAAA, 222::GFP^ (LEU2), ura3-52::prGAL1-CIK1-CC (URA3)* | 2-FIG SUPP 2A |
| 4311 | YEF473 | *MATa, spc105Δ::NAT, leu2Δ-1::Spc105-3A (T235A, T284A, T313A)^101-104, 340-343::AAAA, 222::GFP^ (LEU2), ura3-52::prGAL1-CIK1-CC (URA3)* | 2-FIG SUPP 2A |
| 5738 | YEF473 | *MATa, spc105Δ::NAT, leu2Δ-1::Spc105-4A (T149A, T211A, T235A, T284A)-12XMYC (LEU2), ura3-52::prGal1-CIK1-CC (URA3)* | 2-FIG SUPP 2A |
| 5739 | YEF473 | *MATa, spc105Δ::NAT, leu2Δ-1::Spc105-4A (T149A, T211A, T235A, T284A)-12XMYC (LEU2), ura3-52::prGal1-CIK1-CC (URA3)* | 2-FIG SUPP 2A |
| 5740 | YEF473 | *MATa, spc105Δ::NAT, leu2Δ-1::Spc105-4A (*T172A, T235A, T284A, T313A)*^222::GFP^ (LEU2), ura3-52::prGal1-CIK1-CC (URA3)* | 2-FIG SUPP 2A |
| 5741 | YEF473 | *MATa, spc105Δ::NAT, leu2Δ-1::Spc105-4A (*T172A, T235A, T284A, T313A)*^222::GFP^ (LEU2), ura3-52::prGal1-CIK1-CC (URA3)* | 2-FIG SUPP 2A |
| 4279 | W303 | *MATα, sgo1Δ::HYG, ura3-52::prGAL1-CK1-CC-TAP (URA3)* | 2-FIG SUPP 2A |
| 3727 | YEF473 | *MATa, spc105Δ::NAT, leu2Δ-1::Spc105^709::GFP^ (LEU2), SPC25-mCherry-KAN* | 3-FIG SUPP 1A |
| 3728 | YEF473 | *MATa, spc105Δ::NAT, leu2Δ-1::Spc105^709::GFP^ (LEU2), SPC25-mCherry-KAN* | 3-FIG SUPP 1A |
| 3729 | YEF473 | *MATa, spc105Δ::NAT, leu2Δ-1::Spc105^455::GFP^ (LEU2), SPC25-mCherry-KAN* | 3-FIG SUPP 1A |
| 3730 | YEF473 | *MATa, spc105Δ::NAT, leu2Δ-1::Spc105^455::GFP^ (LEU2), SPC25-mCherry-KAN* | 3-FIG SUPP 1A |
| 3731 | YEF473 | *spc105Δ::NAT, leu2Δ-1::Spc105^222::GFP^ (LEU2), SPC25-mCherry-KAN* | 3-FIG SUPP 1A |
| 3732 | YEF473 | *MATα, spc105Δ::NAT, leu2Δ-1::Spc105^222::GFP^ (LEU2), SPC25-mCherry-KAN* | 3-FIG SUPP 1A |
| 3733 | YEF473 | *MATα, SPC105-GFP-KAN, SPC25-mCherry-KAN* | 3-FIG SUPP 1A |
| 3734 | YEF473 | *MATα, SPC105-GFP-KAN, SPC25-mCherry-KAN* | 3-FIG SUPP 1A |
| 2188 |  | *MATa, spc105Δ::LEU2, his3Δ1::GFP-Spc105 (HIS3), SPC25-mCherry-KAN* | 3-FIG SUPP 1A |
| 2189 |  | *MATa, spc105Δ::LEU2, his3Δ1::GFP-Spc105 (HIS3), SPC25-mCherry-KAN* | 3-FIG SUPP 1A |
| 3640 |  | *MATa, spc105Δ::NAT, his3Δ1::GFP-SPC105 (HIS3), DAD4-mCherry-NAT* | 3-FIG SUPP 1A |
| 3642 | YEF473 | *MATa, spc105Δ::NAT, leu2Δ-1::Spc105^455::GFP^ (LEU2), DAD4-mCherry-NAT* | 3-FIG SUPP 1A |
| 3510 | YEF473 | *MATa, spc105Δ::NAT, leu2Δ-1::Spc105^222::GFP^ (LEU2), DAD4-mCherry-NAT* | 3-FIG SUPP 1A |
| 5015 | YEF473 | *MATa/α, spc105Δ::NAT/SPC105, leu2Δ-1/leu2Δ-1::Spc105-3A (#4-6 in #1-3, T235A, T284A, T313A) RASA(V76, F78::A)^222::GFP^ (LEU2)* | 5-FIG SUPP1C |
| 5016 | YEF473 | *MATa/α, spc105Δ::NAT/SPC105, leu2Δ-1/leu2Δ-1::Spc105-3A (T149A, T172A, T211A, #1-3 in #4-6) RASA (V76, F78::A)^222::GFP^ (LEU2)* | 5-FIG SUPP1C |
| 5076 | YEF473 | *MATa, spc105Δ::NAT1, leu2Δ-1::Spc105-3A (#4-6 in #1-3, T235A, T284A, T313A) RASA (V76, F78::A) ^222::GFP^ (LEU2), BUB3-mCherry-HYG* | 5-FIG SUPP1D |
| 5077 | YEF473 | *MATa, spc105Δ::NAT1, leu2Δ-1::Spc105-3A (#4-6 in #1-3, T235A, T284A, T313A) RASA (V76, F78::A)^222::GFP^ (LEU2), BUB3-mCherry-HYG* | 5-FIG SUPP1D |
| 5339 | YEF473 | *MATa, spc105Δ::NAT1, leu2Δ-1::Spc105-3A* (T149A, T172A, T211A) *RASA (V76, F78::A)^222::GFP^ (LEU2), MAD1-mCherry-HYG, nup60Δ::TRP1* | 5-FIG SUPP1E |
| 5340 | YEF473 | *MATa, spc105Δ::NAT1, leu2Δ-1::Spc105-3A* (T149A, T172A, T211A) *RASA (V76, F78::A)^222::GFP^ (LEU2), MAD1-mCherry-HYG, nup60Δ::TRP1* | 5-FIG SUPP1E |
