# Supplementary material for: The copy-number and varied strengths of MELT motifs in Spc105 balance the strength and responsiveness of the spindle assembly checkpoint
Source: eLife. 2020 Jun 1;9:e55096. doi: 10.7554/eLife.55096 (PMC7292645; doi:10.7554/eLife.55096)
Supplement: Supplementary file 3. [file elife-55096-supp3.docx]

Supplementary file 3: Plasmids used in this study.

| Plasmid (pAJ#) | Origin | Parent | Description |
| --- | --- | --- | --- |
| pAJ212 | Joglekar lab | pRS303 | *prSPC105*+*GFP-SPC105-12MYC (HIS3)* |
| pAJ332 | Joglekar lab | pRS305 | *prSPC105*+Spc105-6A (T149A, T172A, T211A, T235A, T284A, T313A)-12X*MYC*+*trSPC105 (LEU2)* |
| pAJ335 | Joglekar lab | pRS305 | *prSPC105*+Spc105-5A (T149A, T211A, T235A, T284A, T313A)-12X*MYC*+*trSPC105 (LEU2)* |
| pAJ351 | Joglekar lab | pRS305 | *prHIS3-GFP(S65T)-* Spc105 (120-329)-FRB- SV40 NLS *(LEU2)* |
| pAJ406 | Joglekar lab | pRS305 | *prSPC105*+Spc105-5A (T149A, T172A, T211A, T235A, T284A)-12X*MYC*+*trSPC105 (LEU2)* |
| pAJ418 | This study | pRS305 | *prSPC105*+*Spc105^709::GFP^+trSPC105 (LEU2)* |
| pAJ419 | This study | pRS305 | *prSPC105*+*Spc105^455::GFP^+ trSPC105 (LEU2)* |
| pAJ449 | Joglekar lab | pRS305 | *prSPC105*+Spc105^222::GFP^+*trSPC105 (LEU2)* |
| pAJ526 | Joglekar lab | pRS305 | *prSPC105*+Spc105^222::GFP, 101-104, 340-343::AAAA^+*trSPC105 (LEU2)* |
| pAJ551 | Joglekar lab | pRS305 | *prHIS3-GFP(S65T)-*Spc105 (2-329)-FRB-SV40 NLS *(LEU2)* |
| pAJ554 | Joglekar lab | pRS305 | *prSPC105*+Spc105-4A (T149A, T211A, T235A, T284A)-12XMYC+*trSPC105 (LEU2)* |
| pAJ576 | This study | pRS305 | *prSPC105*+Spc105-3A (T149A, T172A, T211A) ^222::GFP, 101-104, 340-343::AAAA^ +*trSPC105 (LEU2)* |
| pAJ577 | This study | pRS305 | *prSPC105*+Spc105-3A (T235A, T284A, T313A) ^222::GFP, 101-104, 340-343::AAAA^ +*trSPC105 (LEU2)* |
| pAJ581 | This study | pRS305 | *prSPC105*+Spc105-5A (T149A, T172A, T211A, T284A, T313A)-12XMYC+*trSPC105 (LEU2)* |
| pAJ606 | This study | pRS305 | *prSPC105*+Spc105-5A (T149A, T211A, T235A, T284A, T313A)^101-104::AAAA^ -12XMYC+*trSPC105 (LEU2)* |
| pAJ607 | This study | pRS305 | *prSPC105*+Spc105-5A (T149A, T172A, T211A, T235A, T284A)^101-104::AAAA^ -12XMYC+*trSPC105 (LEU2)* |
| pAJ608 | This study | pRS305 | *prSPC105*+Spc105-5A (T149A, T172A, T211A, T284A, T313A)^101-104::AAAA^ -12XMYC+*trSPC105 (LEU2)* |
| pAJ609 | This study | pRS305 | *prSPC105*+Spc105-3A (T149A, T172A, T211A) ^222::GFP^ +*trSPC105 (LEU2)* |
| pAJ613 | Joglekar lab | pRS305 | *prSPC105*+Spc105-3A (T235A, T284A, T313A) ^222::GFP^ +*trSPC105 (LEU2)* |
| pAJ630 | This study | pRS305 | *prSPC105*+Spc105-4A (T211A, T235A, T284A, T313A)^222::GFP^+*trSPC105 (LEU2)* |
| pAJ631 | This study | pRS305 | *prSPC105*+Spc105-4A (T172A, T235A, T284A, T313A)^222::GFP^+*trSPC105 (LEU2)* |
| pAJ639 | This study | pRS305 | *prSPC105*+Spc105-3A (T149A, T172A, T211A) ^222::GFP, 101-104::AAAA^ +*trSPC105 (LEU2)* |
| pAJ658 | This study | pAJ305 | *prSPC105+*Spc105-5A (T149A, T172A, T211A, T284A, ME310-311ID)^101-104::AAAA^-12XMYC*(LEU2)* +*trSPC105 (LEU2)* |
| pAJ669 | Storchova lab | pRS306 | *prGAL1-CIK1-CC-TAP (URA3)* |
| pAJ697 | This study | pRS305 | *prSPC105*+ Spc105-3A (#4-6, T149A, T172A, T211A)^222::mCherry, 101-104, 340-343::AAAA^ (*LEU2*) |
| pAJ698 | This study | pRS305 | *prSPC105*+ Spc105-3A (#4-6, , T235A, T284A, T313A)^222::mCherry, 101-104, 340-343::AAAA^ (*LEU2*) |
| pAJ737 | This study | pRS305 | *prSPC105*+ Spc105-3A (#4-6 in #1-3, T235A, T284A, T313A)^222::GFP^ (*LEU2*) |
| pAJ747 | This study | pRS305 | *prSPC105*+ Spc105-3A (T149A, T172A, T211A, #1-3 in #4-6)^222::GFP^ (*LEU2*) +*trSPC105 (LEU2)* |
| pAJ755 | This study | pRS305 | *prSPC105*+ Spc105-3A (#4-6 in #1-3, T235A, T284A, T313A)^222::GFP, 101-104 ::AAAA^ (*LEU2*) |
| pAJ756 | This study | pRS305 | *prSPC105*+ Spc105-3A (T149A, T172A, T211A, #1-3 in #4-6)^222::GFP, 101-104 ::AAAA^ (*LEU2*) +*trSPC105 (LEU2)* |
| pAJ775 | Joglekar lab | pRS305 | *prSPC105*+Spc105^222::GFP, RASA (V76, F78::A)^+*trSPC105 (LEU2)* |
| pAJ782 | This study | pRS306 | *prSPC105*+Spc105^222::GFP, RASA (V76, F78::A)^+*trSPC105 (URA3)* |
| pAJ797 | This study | pRS305 | *prSPC105*+Spc105-3A (T149A, T172A, T211A) ^222::GFP, RASA (V76, F78::A)^ +*trSPC105 (LEU2)* |
| pAJ798 | This study | pRS305 | *prSPC105*+Spc105-3A (T235A, T284A, T313A) ^222::GFP, RASA (V76, F78::A)^ +*trSPC105 (LEU2)* |
| pAJ799 | This study | pRS305 | *prSPC105*+ Spc105-3A (#4-6 in #1-3, T235A, T284A, T313A)^222::GFP, RASA (V76, F78::A)^ +*trSPC105* (*LEU2*) |
| pAJ800 | This study | pRS305 | *prSPC105*+ Spc105-3A (T149A, T172A, T211A, #1-3 in #4-6)^222::GFP, RASA (V76, F78::A)^ (*LEU2*) +*trSPC105 (LEU2)* |
| pAJ818 | Joglekar lab | pSK954 | *prSPC105*+Spc105^222::mCherry^+*trSPC105 (KAN)* |
| pAJ852 | This study | pSK954 | *prBUB1+bub1^T453A, T455A^+2XFKBP12+trBUB1 (KAN)* |
| pAJ867 | This study | pRS305 | *prSPC105*+Spc105 -5A (T149A, M171L, T211A, T235A, T284A, T313A) ^222::GFP, 101-104, 340-343::AAAA^+*trSPC105 (LEU2)* |
| pAJ868 | This study | pRS305 | *prSPC105*+Spc105 -5A (T149A, T172A, T211A, ID232-233ME, T284A, T313A) ^222::GFP, 101-104,340-343::AAAA^+*trSPC105 (LEU2)* |
| pAJ869 | This study | pRS305 | *prSPC105*+Spc105 -5A (T149A, T172A, T211A, T235A, T313A) ^222::GFP, 101-104,340-343::AAAA^+*trSPC105 (LEU2)* |
| pAJ870 | This study | pRS305 | *prSPC105*+Spc105 -5A (T149A, T172A, T211A, T235A, I283L, T313A) ^222::GFP, 101-104, 340-343::AAAA^+*trSPC105 (LEU2)* |
| pAJ896 | This study | pSK954 | *prBUB1+bub1^T453A, T455A^+2XFKBP12+trADH1 (KAN)* |
| pAJ898 | This study | pRS306 | *prSPC105*+Spc105-3A (T149A, T172A, T211A) ^222::GFP, RASA (V76, F78::A)^ +*trSPC105 (URA3)* |
| pAJ904 | This study | pRS305 | *prSPC105*+ Spc105^455::GFP^ MELT1(X6) +*trSPC105* (*LEU2*) |
