# Supplementary figures and images for: The copy-number and varied strengths of MELT motifs in Spc105 balance the strength and responsiveness of the spindle assembly checkpoint
Source: eLife. 2020 Jun 1;9:e55096. doi: 10.7554/eLife.55096 (PMC7292645; doi:10.7554/eLife.55096)

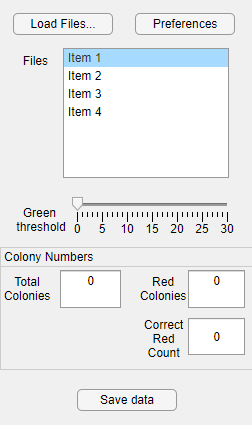

Supplement: Supplementary file 4. [file elife-55096-supp4.mlapp › metadata/appScreenshot.png]
