# Supplementary material for: The copy-number and varied strengths of MELT motifs in Spc105 balance the strength and responsiveness of the spindle assembly checkpoint
Source: eLife. 2020 Jun 1;9:e55096. doi: 10.7554/eLife.55096 (PMC7292645; doi:10.7554/eLife.55096)
Supplement: Transparent reporting form [file elife-55096-transrepform.docx]

***eLife’s* transparent reporting form**

We encourage authors to provide detailed information *within their submission* to facilitate the interpretation and replication of experiments. Authors can upload supporting documentation to indicate the use of appropriate reporting guidelines for health-related research (see [EQUATOR Network](http://www.equator-network.org/%20)), life science research (see the [BioSharing Information Resource](https://biosharing.org/)), or the [ARRIVE guidelines](http://www.plosbiology.org/article/info:doi/10.1371/journal.pbio.1000412) for reporting work involving animal research. Where applicable, authors should refer to any relevant reporting standards documents in this form.

If you have any questions, please consult our Journal Policies and/or contact us: [editorial@elifesciences.org](mailto:editorial@elifesciences.org).

**Sample-size estimation**

- You should state whether an appropriate sample size was computed when the study was being designed
- You should state the statistical method of sample size computation and any required assumptions
- If no explicit power analysis was used, you should describe how you decided what sample (replicate) size (number) to use

Please outline where this information can be found within the submission (e.g., sections or figure legends), or explain why this information doesn’t apply to your submission:

* We did not use explicit power analysis to determine the sample size in each; the size was determined empirically. To ensure reliable inference, we conducted more than two technical replicates and, in most cases, two biological replicates. In imaging experiments, at least 20 fields of cells were collected in each replicate.

* The sample size for each experiment has been noted in legend of the corresponding figure.

* In most cases, the effect size is large enough to be self-evident. Furthermore, we have displayed each data point individually so that the reader can directly assess the effect size and its significance.

**Replicates**

- You should report how often each experiment was performed
- You should include a definition of biological versus technical replication
- The data obtained should be provided and sufficient information should be provided to indicate the number of independent biological and/or technical replicates
- If you encountered any outliers, you should describe how these were handled
- Criteria for exclusion/inclusion of data should be clearly stated
- High-throughput sequence data should be uploaded before submission, with a private link for reviewers provided (these are available from both GEO and ArrayExpress)

Please outline where this information can be found within the submission (e.g., sections or figure legends), or explain why this information doesn’t apply to your submission:

* The number of biological and technical replicates has been noted in the legend for each figure.

* Biological replicates are defined as multiple transformants or segregants of the same genotype. Technical replicates reflect the number of times each experiment was performed. This definition can be found in the Statistical Analysis subsection of Methods (Pages 22-23).

**Statistical reporting**

- Statistical analysis methods should be described and justified
- Raw data should be presented in figures whenever informative to do so (typically when N per group is less than 10)
- For each experiment, you should identify the statistical tests used, exact values of N, definitions of center, methods of multiple test correction, and dispersion and precision measures (e.g., mean, median, SD, SEM, confidence intervals; and, for the major substantive results, a measure of effect size (e.g., Pearson's r, Cohen's d)
- Report exact p-values wherever possible alongside the summary statistics and 95% confidence intervals. These should be reported for all key questions and not only when the p-value is less than 0.05.

Please outline where this information can be found within the submission (e.g., sections or figure legends), or explain why this information doesn’t apply to your submission:

- The details of the statistical methods used in this study are mentioned in the Methods section (Statistical Analysis, Page 22-23).
- We have detailed the data presentation in the figure legends (mean +/- s.e.m. in most cases). The exact p-values indicating statistical significance are displayed on each figure
- In Fig 2A and figure 2-figure supplement 1B, the experiment was performed only twice. Therefore, we only display the mean value in the figure.
- Details of the statistical tests performed are summarized in the ‘Statistical Analysis’ sub-section of the Methods section on pages 22-23.

(For large datasets, or papers with a very large number of statistical tests, you may upload a single table file with tests, Ns, etc., with reference to sections in the manuscript.)

**Group allocation**

- Indicate how samples were allocated into experimental groups (in the case of clinical studies, please specify allocation to treatment method); if randomization was used, please also state if restricted randomization was applied
- Indicate if masking was used during group allocation, data collection and/or data analysis

Please outline where this information can be found within the submission (e.g., sections or figure legends), or explain why this information doesn’t apply to your submission:

Not applicable.

**Additional data files (“source data”)**

- We encourage you to upload relevant additional data files, such as numerical data that are represented as a graph in a figure, or as a summary table
- Where provided, these should be in the most useful format, and they can be uploaded as “Source data” files linked to a main figure or table
- Include model definition files including the full list of parameters used
- Include code used for data analysis (e.g., R, MatLab)
- Avoid stating that data files are “available upon request”

Please indicate the figures or tables for which source data files have been provided:

We used custom Graphical User Interfaces written in Matlab for quantify the fluorescence intensity from kinetochore proteins or kinetochore-localized SAC proteins. These GUI’s were designed for use in-house; they require a specific work-flow, and they are not user-friendly. Therefore, we have not included the code for these image analysis programs with the submission. The straight-forward image analysis methodologies that these GUIs implement have been described in detail in previous work from the lab. It is also described in the Methods section (Microscopy and Image analysis section, Chromosome Loss quantification).
