# Supplementary material for: The copy-number and varied strengths of MELT motifs in Spc105 balance the strength and responsiveness of the spindle assembly checkpoint
Source: eLife. 2020 Jun 1;9:e55096. doi: 10.7554/eLife.55096 (PMC7292645; doi:10.7554/eLife.55096)
Supplement: Peer review history

# Decision letter

**DOI:** 10.7554/eLife.55096.sa1

**Affiliations:** Institute of Cancer Research ResearchUnited Kingdom; Institute of Cancer Research ResearchUnited Kingdom; University of CopenhagenGermany

In the interests of transparency, eLife publishes the most substantive revision requests and the accompanying author responses.

**Acceptance summary:**

The presence of multiple MELT motifs at each kinetochore is puzzling since only one is strictly required for a checkpoint. This paper nicely demonstrates how the different affinities of the multiple MELT motif receptors contribute to the responsiveness of the spindle assembly checkpoint to microtubule attachment.

**Decision letter after peer review:**

Thank you for submitting your article "The number and activity of MELT motifs in Spc105 balance the strength and responsiveness of the Spindle Checkpoint" for consideration by *eLife*. Your article has been reviewed by three peer reviewers, including Jon Pines as the Reviewing Editor and Reviewer #1, and the evaluation has been overseen by Anna Akhmanova as the Senior Editor. The following individual involved in review of your submission has agreed to reveal their identity: Jakob Nilsson (Reviewer #3).

The reviewers have discussed the reviews with one another and the Reviewing Editor has drafted this decision to help you prepare a revised submission.

Summary:

This is a comprehensive and insightful analysis of the role of MELT motifs in modulating the strength and responsiveness of the spindle assembly checkpoint (SAC). The authors use a variety of well-designed assays to conclude that the number of sequence of the MELT motifs is optimised for a strong but responsive SAC, and that the mechanism behind this is a difference the dephosphorylation of Bub1 and the MELT motifs by PP1 recruited to Spc105. High affinity MELT motifs are more resistant to dephosphorylation through binding more strongly to Bub3, which interferes with access by PP1.

Essential revisions:

1) The authors exclude the role of Sgo1 recruitment in giving differences between the benomyl platings of MELT#1-3 and MELT-#4-6. However, they do not show that the difference is due to the strength of the SAC. They should delete Mad1 in these strains and see if the difference is gone. An alternative possibility is that the loss of MELT motifs #1-3 slightly disrupts kinetochore function, giving rise to a SAC-independent increase in chromosome mis-segregation, which would give a benomyl defect and is supported by their findings in Figure 2F.

2) The longer doubling times from the *spc105^RASA^bub1^453A,455A^* could be due to aneuploidy of the strain, not SAC signaling. The Rosenberg et al., 2011, paper mentions that the *spc105^RASA^* strains have an increase in chromosome mis-segregation. To conclude that this delay is due to defects in SAC silencing, the authors must make a deletion of Mad1 and show that the delay is no longer present.

3) In Figure 6D, is this delay dependent on the spindle checkpoint? Is it abrogated in a Mad1 deletion?

4) Clearly the capacity of Bub1 to bind Mad1 is critical for SAC signaling and it is unclear to me if the different MELTs differ in their ability to form the Bub1-Mad1 complex for instance due to Mps1 activity? Have the authors looked more carefully at Mad1 levels in the different Spc105 variants?

# Reply: Author response

**DOI:** 10.7554/eLife.55096.sa2

Essential revisions:

1) The authors exclude the role of Sgo1 recruitment in giving differences between the benomyl platings of MELT#1-3 and MELT-#4-6. However, they do not show that the difference is due to the strength of the SAC. They should delete Mad1 in these strains and see if the difference is gone. An alternative possibility is that the loss of MELT motifs #1-3 slightly disrupts kinetochore function, giving rise to a SAC-independent increase in chromosome mis-segregation, which would give a benomyl defect and is supported by their findings in Figure 2F.

The suggested experiment would support the notion that the difference between MELT#1-3 and MELT#4-6 is mainly due to a difference in the SAC signaling rather than a specific, unknown function of the first three MELT motifs. However, we want to point out that Mad1 deletion is lethal in benomyl-containing media even with wild-type Spc105. Therefore, it stands to reason that it will be lethal with Spc105^MELT#1-3^. Based on what we known, the most likely reason for the significantly higher chromosome missegregation rate in the MELT#4-6 strain is the reduced Bub1 recruitment to the kinetochore (shown in new Figure 2—figure supplement 3C), which in turn is likely to reduce Sgo1 recruitment. Confirming this hypothesis will require a direct biochemical confirmation of this hypothesis. Instead, we have included correlative data showing that Sgo1-GFP recruitment to prometaphase kinetochores is lower in strains expressing Spc105MELT^#4-6^ than in strains expressing Spc105^MELT#1-3^. We state that this likely explanation as follows.

Subsection “Spc105 mutants containing only weak MELT motifs experience high rates of chromosome missegregation during unperturbed cell division”: “The weaker SAC signaling in this strain is observed only when the SAC is activated by a small number of unattached kinetochores (Figure 2—figure supplement 3B). […] The lower Sgo1 recruitment ultimately impairs chromosome biorientation, and therefore elevates chromosome missegregation frequency.”

2) The longer doubling times from the spc105^RASA^ bub1^453A,455A^ could be due to aneuploidy of the strain, not SAC signaling. The Rosenberg et al., 2011, paper mentions that the spc105^RASA^ strains have an increase in chromosome mis-segregation. To conclude that this delay is due to defects in SAC silencing, the authors must make a deletion of Mad1 and show that the delay is no longer present.

We completely agree. We qualify our conclusion by stating the missing data as follows.

Subsection “PP1-mediated dephosphorylation of Bub1 contributes to SAC silencing, but this silencing mechanism is not as efficient as PP1-mediated dephosphorylation of MELT motifs”: “The *spc105^RASA^ bub1^453A, 455A^* double mutant exhibited a longer doubling time in YPD, which is suggestive of a SAC silencing defect arising from the lack of PP1 recruitment by Spc105 (Figure 4C, scatter plot on the right). Thus, PP1-mediated dephosphorylation of several phosphorylated residues on Bub1 suppresses Mad1 recruitment and thereby contributes to the responsiveness of the SAC to silencing.”

3) In Figure 6D, is this delay dependent on the spindle checkpoint? Is it abrogated in a Mad1 deletion?

This is a good point. We qualify our conclusion as follows:

Subsection “Engineering an Spc105 allele for optimal SAC signaling, silencing, and error correction”: “The higher doubling time in this case is likely due to an SAC silencing defect, although this needs to be confirmed experimentally. Importantly, the slower growth rate is accompanied by improved chromosome segregation.”

4) Clearly the capacity of Bub1 to bind Mad1 is critical for SAC signaling and it is unclear to me if the different MELTs differ in their ability to form the Bub1-Mad1 complex for instance due to Mps1 activity? Have the authors looked more carefully at Mad1 levels in the different Spc105 variants?

We did not quantify Mad1 recruitment in the Spc105 variants in this study. However, we examined this relationship in detail in our previous study (Figures 3F, 4A-B in Aravamudhan et al., MBoC). First, we found that Mad1 recruitment by Spc105 with either MELT#2 or MELT#6 as the active MELT motifs was not significantly different in cells treated with nocodazole. In the second experiment, we analyzed the dependence between Bub1 and Mad1 recruitment. We found that Mad1 recruitment is mainly limited by the abundance of Bub1, because Bub1 over-expression is sufficient to increase Mad1 recruitment in nocodazole-treated cells. These data further suggest that the affinity and number of MELT motifs, and Bub1 abundance, are the two main factors controlling the strength of SAC signaling. Under normal expression level of Bub1, Mad1 recruitment does not appear to be affected by the amino acid sequence of the underlying MELT motif. We include this information in the revised manuscript as follows.

Subsection “The number of high affinity MELT motifs in Spc105 and Bub1 expression together determine SAC responsiveness to silencing mechanisms”: “These experiments along with our prior observations showing that Bub1 abundance primarily limits Mad1 recruitment to the cell show that the low expression level of Bub1 limits the recruitment of Bub3-Bub1 and Mad1-Mad2 to unattached yeast kinetochores and thus primarily limits SAC strength. The number and affinity of MELT motifs determines both the strength and the responsiveness of the SAC to PP1-mediated silencing.”
